# The effects of pegylated interferon-α and ribavirin on liver and serum concentrations of activin-A and follistatin in normal Wistar rat: a preliminary report

**DOI:** 10.1186/s13104-015-1253-2

**Published:** 2015-06-26

**Authors:** Bassem Refaat, Adel Galal El-Shemi, Ahmed Mohammed Ashshi

**Affiliations:** Laboratory Medicine Department, Faculty of Applied Medical Sciences, Umm Al-Qura University, Al Abdeyah, PO Box 7607, Makkah, KSA; Department of Pharmacology, Faculty of Medicine, Assiut University, Assiut, Egypt

**Keywords:** Activin-A, Follistatin, Pegylated interferon-α, Ribavirin, Liver

## Abstract

**Background:**

Activin-A and follistatin regulate the liver and the immune system.

**Aims:**

To measure the effects of treatment with pegylated-interferon-α (Peg-IFN-α) and ribavirin on the concentrations of mature activin-A and follistatin in serum and liver tissue homogenates in rats.

**Methods:**

A total of 28 male Wistar rats were divided equally into four groups as follow: ‘Control group’ (n = 7), ‘PEG only group’ consisted of those that only received a weekly injection of Peg-IFN-α (6 µg/rat) for 4 weeks, ‘RBV only group’ received ribavirin only (4 mg/rat/day) orally for 35 days and the last group received both Peg-IFN-α and ribavirin ‘PEG & RBV group’. The concentrations of candidate proteins in serum and liver samples were measured using ELISA.

**Results:**

Pegylated-interferon-α decreased activin-A and increased follistatin significantly in serum and liver of ‘PEG only’ and ‘PEG & RBV’ groups compared with the ‘Control’ and ‘RBV only’ groups (P < 0.05). There was no significant difference between the ‘RBV only’ and ‘Control’ groups (P > 0.05) in the concentrations of candidate proteins. A significant positive correlations between serum and liver activin-A (r = 0.727; P = 0.02 × 10^−3^) and follistatin (r = 0.540; P = 0.01) was also detected.

**Conclusion:**

Pegylated-interferon-α modulates the production of activin-A and follistatin by the liver, which is reflected and can be detected at the serum level. Further studies are needed to explore the role of Peg-IFN-α based therapy on the production of activins and follistatin by the liver and immune cells.

## Background

Infection with hepatitis C virus (HCV) is a global health problem and it is a leading cause for the development of liver fibrosis, cirrhosis and hepatocellular carcinoma [1]. Although new antiviral drugs have been developed, the treatment of chronic hepatitis C (CHC) could still be based on a weekly injection of pegylated interferon-α (Peg-IFN-α)-2a or -2b plus a daily weight-based dose of ribavirin depending on the progression of liver damage and the presence of other extrahepatic manifestations [2–5]. Furthermore, the new directly acting antiviral drugs are expensive and therefore, Peg-IFN-α based therapy could be an affordable alternative approach for treatment naïve patients with no liver cirrhosis and/or for those living in developing countries and for whom access to the new therapy is not definite due to the high cost [6, 7].

The development of hepatic complications following infection with HCV is due the promotion of adaptive immune response by activating T helper (Th)-2 pathway [8, 9]. IFN-α alters the immune response in patients with CHC from Th2 to a Th1 mediated pattern, which favours the eradication of the virus [10, 11]. IFN-α induces Th1 response through the modulation of several cytokines including IFN-γ, tumour necrosis factor (TNF)-α, interleukins (IL) and transforming growth factor (TGF)-β by the hepatocyte and immune cells [12–14].

Activins are members of the TGF-β family and their biological activities are tightly regulated by their binding protein follistatin [15]. Activin-A and follistatin are expressed by the hepatocyte and have been described as major regulators of liver biology, liver regeneration and liver pathology [16]. Additionally, they play an important role in the regulation of the immune system and the pathogenesis of inflammatory and fibrotic human diseases [17].

Activin-A and follistatin have been proposed as diagnostic/prognostic markers for a variety of liver diseases since pathological alterations in their serum concentrations have been documented in several liver pathologies, including viral hepatitis B and C, and they correlate with the severity of the diseases [18, 19]. We have previously demonstrated that both CHC and Peg-IFN-α based therapy modulate the serum concentrations of activin-A and follistatin [20, 21]. Hence, the present pilot study was conducted to test our hypothesis that Peg-IFN-α alters the serum concentrations of these proteins by regulating their production in the liver.

## Methods

### Drugs

Pegylated interferon-α-2a (Pegasys^®^, Hoffmann-La Roche, Nutley, NJ, USA) was used. The ready to use syringe contains 180 µg/0.5 ml. Ribavirin capsules (Viracure^®^, 6th October Pharm, Egypt) were used and each capsule contains 400 mg of ribavirin.

### Study design

All experimental protocols were approved by the Committee for the Care and Use of Laboratory Animals at Umm Al-Qura University and were in accordance with the EU Directive 2010/63/EU for animal experiments.

A total of 28 male Wistar rats weighing 250–300 g were used. All animals received humane care during the study protocol and during euthanasia. The animals were divided equally into four groups as follow: the first group included seven rats that served as ‘Control group’, the second group consisted of those that only received Peg-IFN-α ‘PEG only’ group, the third received ribavirin monotherapy ‘RBV only’ group and the last group consisted of rats that received both Peg-IFN-α and ribavirin dual therapy ‘PEG & RBV’ group.

The study duration was 5 weeks. Peg-IFN-α-2a was prepared as previously reported by diluting the content of a full syringe (0.5 ml) in normal saline to prepare a final volume of 10 ml and the final concentration was 18 µg/ml [22]. Each rat in the ‘PEG only’ and ‘PEG & RBV’ groups received a weekly subcutaneous injection of 0.33 ml (6 µg/rat/week) for a total of 4 injections/rat. One capsule of ribavirin (400 mg) was dissolved in 50 ml saline and each rat in the ‘PEG & RBV’ and ‘RBV only’ groups received 0.5 ml (4 mg)/day orally using a feeding syringe for the whole length of the study similar to the highest dose of the drug recommended for human during CHC treatment [12 mg/kg (1,200 mg for body weight ≥75 kg)] [22]. Following four injections, the rats were euthanised in the fifth week at the same day the 5th injection would have been given. Ribavirin was continued till the day before euthanasia.

### Types of sample

All rats were euthanised on the same day under diethyl ether anaesthesia (Fisher Scientific UK Ltd, Loughborough, UK) a week after the last injection and 4 ml of blood were collected in plain tube immediately after cutting the vena cava. Blood samples were centrifuged for 20 min and the collected serum was stored in −20°C for routine biochemistry and measuring the concentrations of activin-A and follistatin in serum.

A specimen from the middle lobe of the liver (2 g) was obtained from each animal and was immediately used for protein extraction using 6 ml of RIPA lysis buffer containing protease inhibitors (Santa-Cruz Biotechnology Inc, CA, USA) and electrical homogeniser. All samples were centrifuged at 14,000 rpm for 30 min and small aliquots (0.5 ml) of the resultant supernatant were placed in Eppendorf tubes and stored in −20°C till processed to measure the levels of candidate proteins in liver homogenates using ELISA.

### Measurement of extracted protein concentrations

The concentrations of the total proteins extracted from the liver specimens were measured using the BioSpec-nano spectrophotometer (Shimadzu Corporation, Tokyo, Japan) at 280 OD. All protein samples were diluted using normal saline to make a final concentration of 500 µg/ml.

### Enzyme linked immunosorbent assay (ELISA)

Enzyme linked immunosorbent assay was used for the quantitative measurement of activin-A and follistatin (R&D systems, Minneapolis, USA) in serum and liver homogenates samples. All samples were processed in duplicate and according the manufacturer’s instructions. The optical density of the plates was measured within 10 min using a plate reader at 450 nm and correction at 560 nm as recommended by the manufacturer.

The lowest detection limit of activin-A by the used kit was 3.7 pg/ml and the upper limit was 1,500 pg/ml as reported by the manufacturer. The intra-assay and inter-assay precisions of the kit were 4.3 and 5.8%, respectively. The kit could cross react by 0.2 and 0.45% with inhibin-A and activin-AB, respectively. The detection range of the follistatin kit was 250–16,000 pg/ml and the minimum detectable dose was 83 pg/ml.

### Statistical analysis

Statistical analysis of the results was performed using SPSS version 16. Normality and homogeneity of data were assessed with the Kolmogorov and Smirnoff test and Levene test, respectively. One way ANOVA followed by Tukey post hoc test or Kruskal–Wallis followed by Dunn’s post hoc test were used to compare between the different groups depending on the data homogeneity. Correlations were determined using Pearson’s test. P value < 0.05 was considered significant.

## Results

### Results of routine biochemistry

There was no significant difference (P > 0.05) using one way ANOVA between the different study groups in body weight, liver weight, liver enzymes, metabolic profile and renal function parameters (Table 1).

**Table 1:** Mean ± SD of body weight, total liver weight, liver enzymes, albumin, renal function and metabolic parameters in the different study groups

	Control	PEG-IFN Only	PEG-IFN & Ribavirin	Ribavirin Only
Body weight (g)	221.57 ± 20.01	231.97 ± 23.01	218.42 ± 13.64	230.1 ± 22.2
Liver weight (g)	11.04 ± 1.26	10.54 ± 1.52	10.27 ± 0.44	10.23 ± 1.5
ALT (U/l)	60.4 ± 16.2	68.8 ± 18.6	53.9 ± 19	64.1 ± 11.5
AST (U/l)	92.4 ± 24.2	129.8 ± 46.7	119 ± 21.6	133 ± 49.8
Albumin (g/dl)	3.4 ± 0.4	3.3 ± 0.5	3.7 ± 0.3	3.5 ± 0.5
Creatinine (mg/dl)	0.22 ± 0.03	0.2 ± 0.06	0.2 ± 0.03	0.19 ± 0.03
Urea (mg/dl)	47.6 ± 5.1	52.3 ± 4	56.6 ± 9.5	47.3 ± 5.8
BUN (mg/dl)	22.2 ± 2.4	24.4 ± 1.9	26.3 ± 4.4	22 ± 2.7
Glucose (mg/dl)	110.7 ± 12.7	118.9 ± 23.1	112.6 ± 12.7	114.5 ± 16.1
Triglycerides (mg/dl)	77.3 ± 20.3	68.3 ± 13.9	63.2 ± 9.5	85 ± 28.5
Cholesterol (mg/dl)	45 ± 7.2	58.5 ± 12.4	62.2 ± 14.5	64.8 ± 9.7
HDL-C (g/dl)	36.2 ± 9.2	44.3 ± 1.2	52.8 ± 7.8	53.1 ± 7.7
LDL-C (g/dl)	6.6 ± 1.7	8.14 ± 2.33	7.1 ± 2.5	6.3 ± 1.55

### Concentrations of serum and liver activin-A and follistatin

Administration of Peg-IFN-α-2a for 4 weeks significantly decreased the concentrations of activin-A and significantly increased the concentrations of follistatin at both serum and liver levels of the ‘PEG only’ and ‘PEG & RBV’ groups compared with ‘Control’ group. Furthermore, significant differences were detected between ‘PEG & RBV’ and ‘PEG only’ in serum, but not liver, concentrations of candidate proteins (Table 2).

**Table 2:** Mean ± SD of serum and liver concentrations of activin-A and follistatin in the different groups

	Activin-A (pg/ml)		Follistatin (pg/ml)	
	Serum	Liver	Serum	Liver
Control	166.5 ± 41.7	216.1 ± 45.7	128.9 ± 40.9	72.6 ± 22.1
PEG only	92.08 ± 15.3^a^	115.2 ± 37.5^a^	354.15 ± 91.1^a^	157.4 ± 28.5^a^
PEG & RBV	56.5 ± 10.9^a, b^	112.4 ± 41.6^a^	212.4 ± 40.4^a, b^	134.4 ± 38.6^a^
RBV only	174.7 ± 35.8^b, c^	281.8 ± 84.2^b, c^	98.9 ± 33.4^b, c^	82.2 ± 23.5^b, c^

^a^<0.05 compared to control.

^b^<0.05 compared to PEG only.

^c^<0.05 compared to PEG & RBV group.

**Figure 1:**
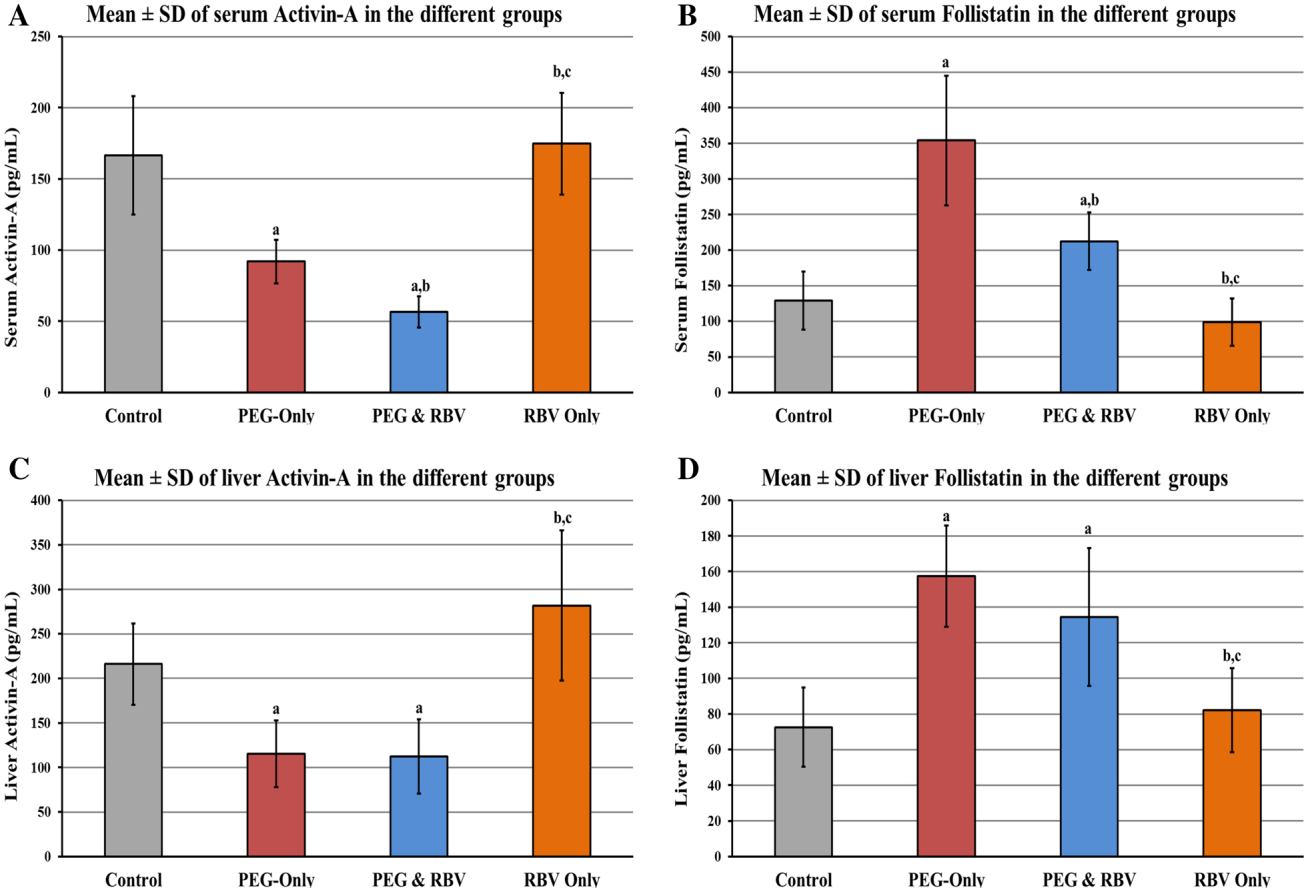
Mean ± SD of **A** serum activin-A, **B** liver activin-A, **C** serum follistatin and **D** liver follistatin in the different study groups (*a* = P < 0.05 compared to control, *b* = P < 0.05 compared to PEG-only group, *c* = P < 0.05 compared to PEG & RBV group).

There was no significant difference in the concentrations of candidate proteins between the ‘RBV only’ and ‘Control’ groups at the serum and liver levels. However, a significant difference was detected between ‘RBV only’ compared with ‘PEG only’ and ‘PEG & RBV’ groups in serum and liver concentrations of activin-A and follistatin (Figure 1).

### Correlations between serum and liver activin-A and follistatin

**Figure 2:**
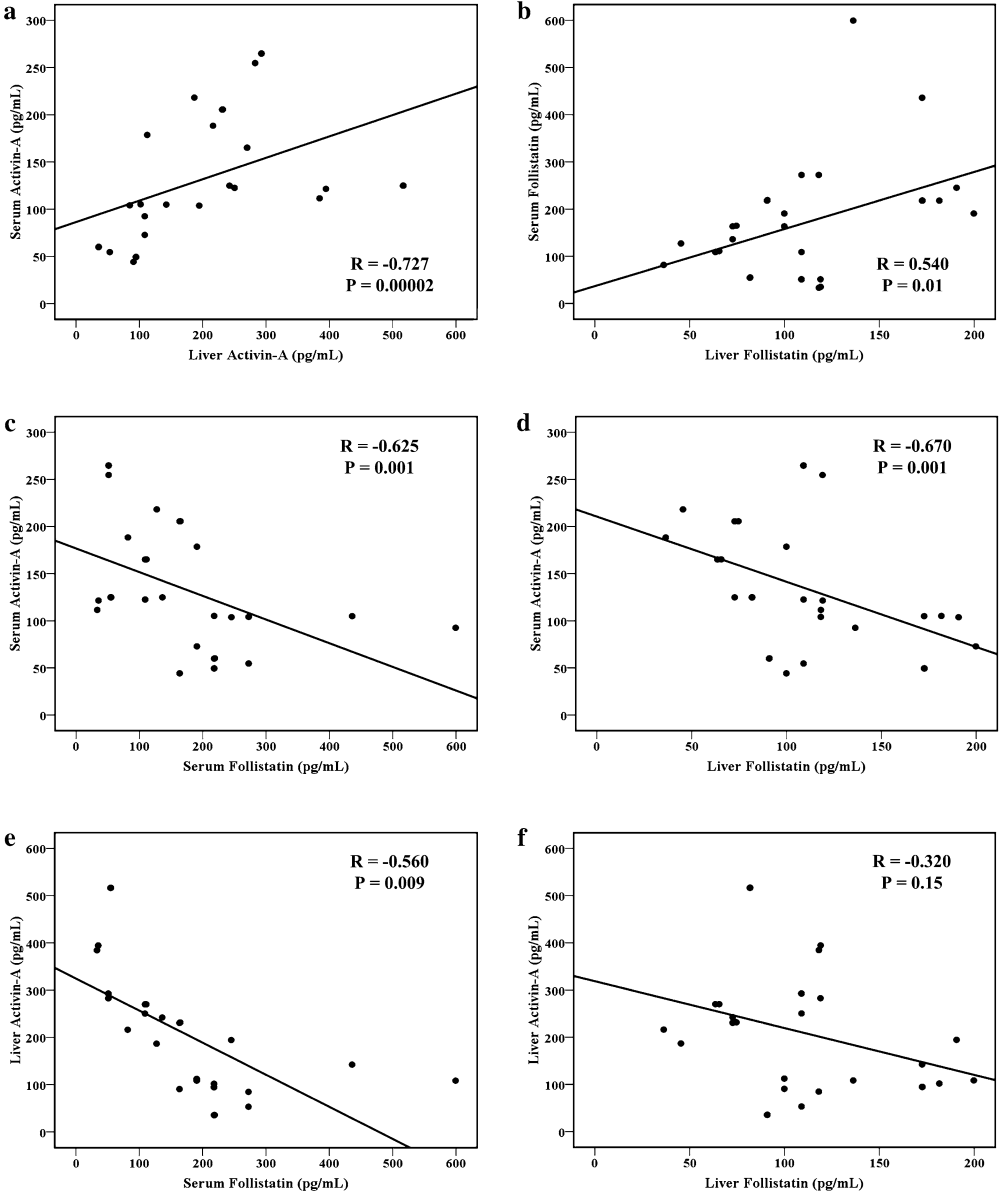
Correlation between **a** serum and liver activin-A, **b** serum and liver follistatin, **c** serum activin-A and serum follistatin, **d** serum activin-A and liver follistatin, **e** liver activin-A and serum follistatin and **f** liver activin-A and follistatin by Pearson’s correlation test.

A significant positive correlation between serum and liver concentrations of activin-A (r = 0.727, P = 0.02 × 10^−3^) and follistatin (r = 0.540, P = 0.01) was observed. Serum activin-A also correlated negatively and significantly with serum follistatin (r = −0.625, P = 0.001) and liver follistatin (r = −0.674, P = 0.001). Additionally, a significant negative correlation was seen between liver activin-A and serum follistatin (r = −0.560, P = 0.009). However, there was a non-significant correlation between liver activin-A and liver follistatin (Figure 2).

## Discussion

To the best of our knowledge, this is the first study to report the effect of Peg-IFN-α and ribavirin on serum and liver concentrations of activin-A and follistatin in experimental animal model. Our results demonstrated a significant decrease in activin-A and significant increase in follistatin at the serum and liver levels in ‘PEG only’ and ‘PEG & RBV’ groups compared with ‘Control’ and ‘RBV only’ groups. Furthermore, our results showed significant correlations between serum and liver activin-A and follistatin.

Our findings suggest that Peg-IFN-α ± ribavirin acts on the liver to modulate the production of activin-A and follistatin, and the alteration in liver concentrations of both proteins following Peg-IFN-α is reflected and detected in serum.

Pegylated-interferon-α alters the immune response in patients with CHC from Th2 to a Th1 mediated pattern to eradicate the viral infection [8]. Activin-A induces deviation of immune responses toward a type 2 phenotype, its serum concentrations increase dramatically in patients with CHC and they correlate with the serum levels of IL-6, TNF-α and the severity of liver damage associated with CHC [20, 23]. Another recent study has also shown that serum activin-A and follistatin are modulated during the treatment of CHC with Peg-IFN-α based therapy and that their levels return to normal in the responder group [21]. Hence, we have hypothesised that activin-A and follistatin are potential target for Peg-IFN-α based therapy [20, 21, 24].

This hypothesis could further be supported by the findings of several other reports. For instant, the expression of toll like receptors-2 and 4, which increases significantly following Peg-IFN-α based therapy in patients with CHC, are potent regulators of the release of activin-A [25–27]. Additionally, the production of TNF-α by the natural killer cells increases and, serum IL-6 and IL-10 decreases following Peg-IFN-α [13, 28]. These cytokines have also been shown to be regulated by activin-A [29]. Moreover, the release of IFN-γ, which plays an important role in controlling CHC following Peg-IFN-α based therapy, is regulated by activin-A [30, 31].

The current observations of significant alterations in serum and hepatic activin-A and follistatin following the administration of Peg-IFN-α ± RBV provide additional support for the aforementioned hypothesis and they are in agreement with our previous findings regarding the effects of Peg-INF-α based therapy on serum activin-A and follistatin during the treatment of CHC [21, 24]. Additionally, the observed significant positive correlations between liver and serum concentrations of activin-A and follistatin, suggest that the liver is a major source of these proteins in serum and alteration in the hepatic production of these proteins following injection of Peg-IFN-α is reflected and detected at the serum level. Further studies are required to illustrate the mechanism(s) by which Peg-IFN-α regulate the production of activin-A and follistatin by the liver.

Although follistatin is regarded as the activin binding protein [32], it appears that the production of follistatin is not only driven by activin during inflammation [33, 34]. The synthesis and secretion of follistatin are also modulated by other cytokines, including IL-1b, TNF-α and IFN-γ [35, 36]. The physiological and pathological activities of activin-A are usually antagonised by follistatin at the cellular and serum levels [32]. Our results agree with these findings as they have shown a significant increase in hepatic and serum concentrations of follistatin following treatment with Peg-IFN-α, suggesting that the drug controls the activities of activin-A by decreasing its production and increasing its binding protein in liver and serum.

Ribavirin monotherapy is not effective in the treatment of CHC and a number of studies have suggested that strong antiviral activity is only seen when RBV is combined with either IFN-α or Peg-IFN-α. Therefore, a synergism between Peg-IFN-α and RBV was suggested and has been shown in vitro [37]. Additionally, it has been suggested that RBV may have immune modulatory activities, including the regulation of macrophage and T helper cells produced cytokines, modulation of the Th1/Th2 subset balance and the enhancement of the expression of interferon stimulated genes [38].

Our results support the previous findings as they have demonstrated a significant decrease in serum activin-A and significant increase in serum follistatin in ‘PEG & RBV’ group compared with ‘PEG only’ and ‘RBV only’ groups. However, these synergistic alterations were only detected at the serum level, suggesting that co-therapy with Peg-IFN-α and ribavirin modulate the production of the candidate molecules at other organs/systems beside the liver that affect the concentrations of these proteins at the serum level.

The biological activities of activins are tightly regulated by follistatin since the binding of activin to follistatin is almost irreversible [15]. Serum activin is commonly bound with the long form follistatin (FS-315) [39], while the short form (FS-288) has high affinity for cell membrane activins [40]. The currently available ELISA kits for the detection of activin-A and follistatin cannot distinguish between the free and bound forms of both proteins. Furthermore, the follistatin kit measures both the long and short forms. Therefore, the reported results in our study are shown at the level of total activin-A and follistatin and the development of ELISA kits that measure the free form of both proteins would expose precisely the effects of Peg-IFN-α on the activity of both proteins.

A limitation of our study that we did not localize the expression of the candidate molecules using other techniques (e.g. immunohistochemistry), which would have revealed the cellular origin of both proteins in the liver. Furthermore, we did not confirm the protein results at the gene level using quantitative PCR. Nevertheless, activins are dimer proteins that are consisted of two β-subunits and therefore both immunohistochemistry and quantitative PCR would only reveal alteration in the expression of activin βA-subunit and not the mature protein [15]. Additionally, this was a phase 1 study and we plan to conduct further studies to measure the effect(s) of Peg-IFN-α on the expression of candidate proteins in a rat model of liver fibrosis and cirrhosis.

## Conclusion

In conclusion, Peg-IFN-α modulates the production of activin-A and follistatin by the liver, which appears to be a major source of serum activin-A and follistatin. Alterations in the concentrations of these proteins in the liver are reflected and can be detected at the serum level. Further studies are needed to explore the role of Peg-IFN-α based therapy on the production of activins and follistatin by the liver and immune cells.
